# Rising incidence of breast cancer among female cancer survivors: implications for surveillance

**DOI:** 10.1038/sj.bjc.6604816

**Published:** 2008-12-09

**Authors:** I Soerjomataram, W J Louwman, L E M Duijm, J W W Coebergh

**Affiliations:** 1 Department of Public Health, Erasmus MC, PO Box 2040, 3000 CA Rotterdam, The Netherlands; 2 Comprehensive Cancer Centre South, PO Box 231, 5600 AE Eindhoven, The Netherlands; 3 Department of Radiology, Catharina Hospital, PO Box 1350, 5602 ZA Eindhoven, The Netherlands

**Keywords:** female survivors, second primary cancer, breast neoplasm, trend

## Abstract

The number of female cancer survivors has been rising rapidly. We assessed the occurrence of breast cancer in these survivors over time. We computed incidence of primary breast cancer in two cohorts of female cancer survivors with a first diagnosis of cancer at ages 30+ in the periods 1975–1979 and 1990–1994. Cohorts were followed for 10 years through a population-based cancer registry. Over a period of 15 years, the incidence rate of breast cancer among female cancer survivors increased by 30% (age-standardised rate ratio (RR-adj): 1.30; 95% CI: 1.03–1.68). The increase was significant for non-breast cancer survivors (RR-adj: 1.41, 95% CI: 1.04–2.75). During the study period, the rate of second breast cancer stage II tripled (RR-adj: 3.10, 95% CI: 1.73–5.78). Non-breast cancer survivors had a significantly (*P* value=0.005) more unfavourable stage distribution (62% stage II and III) than breast cancer survivors (32% stage II and III). A marked rise in breast cancer incidence among female cancer survivors was observed. Research to optimise follow-up strategies for these women to detect breast cancer at an early stage is warranted.

Breast cancer is the most common cancer among women in general, but also among women who were previously diagnosed with any type of cancer (Mariotto *et al*, 2007). During the past three decades, a fourfold increase in the incidence of contralateral breast cancer has been reported, which is much higher than that of first primaries (Louwman *et al*, 2008). However, in the same period, a 9% decrease in the incidence of second breast cancer among former breast cancer patients was reported in the United States of America (Yu *et al*, 2006). The increasing prevalence of patients ever diagnosed with cancer should theoretically result in an increase in the incidence of new primary cancer (Fraumeni *et al*, 2006), that is, breast cancer among cancer survivors. Changes in female reproductive behaviour and lifestyle, that underlie the increasing trend of first breast cancer, may also affect the increased risk of a second breast cancer (Dignam *et al*, 2003; Largent *et al*, 2007). Furthermore, cancer survivors are exposed to additional carcinogenic factors such as high-dose radiation for the first cancer (van Leeuwen and Travis, 2005). Using data from a long-standing cancer registry in southern Netherlands, we investigated the incidence of breast cancer among cancer survivors since 1975. We assessed the change in incidence of a second breast cancer over time according to age, stage and type of treatment of the first cancer.

## Materials and methods

Data on cancer patients were obtained from the Eindhoven Cancer Registry (ECR), located in the south of Netherlands. This is a population-based registry with follow-up data since 1970, including clinical aspects such as stage and initial treatment. The cancer registry regularly receives lists of new cancer cases from the pathology and haematology departments in the region. In addition, lists of all hospitalised cancer patients were obtained, comprising data from hospital medical records. In the ECR, active follow-up of vital status is conducted through linkage with municipal population registries and the Central Bureau for Genealogy. Eindhoven Cancer Registry is a long-standing cancer registry with high-quality data, as indicated by the high proportion of histologically verified cases. (Curado *et al*, 2007) Furthermore, the proportion of new cases identified through death certificates or autopsy only is 0%, indicating efficient case finding (Curado *et al*, 2007). The coverage area of the registry in the south of the Netherlands has gradually increased, covering about 0.9 million people between 1975 and 1985 and over 2 million people since 1988.

### Data analysis

The change in breast cancer incidence among cancer survivors over time was calculated using the fixed inception cohort method (Yu *et al*, 2006). We defined two patient cohorts: women diagnosed with a primary cancer between 1975 and 1979 and those diagnosed between 1990 and 1994. We included all cancer types diagnosed in women aged 30 years or more within the given periods, excluding premalignant or *in situ* cancer and basal cell carcinoma of the skin. Only patients who survived 6 months or longer were included in the cohort. The rules for multiple primary cancers from the International Agency for Cancer Research were used (Working Group Report, 2005). Based on these rules, a person can have only one cancer per organ or pair of organs except when multiple tumours within an organ have a different histology. For breast cancer, two tumours of different laterality but of the same morphology are registered separately (2005). The follow-up time extended from the date of the initial cancer diagnosis to the date of a second cancer, death, loss to follow-up or end of the study, whichever occurred first. We applied a 10-year follow-up for each patient cohort. Thus, the 1975–1979 cohort was followed until 1989 and the 1990–1994 cohort until 2004. Person-years contributed by each survivor were calculated to compute incidence rates per 100 000 person-years. Adjustment for age was performed by calculating predicted rates of expected new breast cancer incident cases, assuming identical age composition (entered in 5-year age categories) of the first and second cohort. We used Poisson regression using Proc genmod in SAS. To compare the two periods, rate ratios of breast cancer and their 95% confidence intervals (95% CI) were computed taking the earlier cohort (those diagnosed with first cancer between 1975–1979) as reference (Breslow and Day, 1987).

To identify the factors causing differences in rates over time, we stratified according to type of first primary cancer (breast and non-breast cancer) and treatment of the first primary (surgery, radiotherapy with or without surgery, systemic therapy with or without surgery, radiotherapy and systemic therapy with or without surgery and no therapy).

Finally, incidence rates of breast cancer in female survivors were calculated stratifying by age (30–49 years, 50–74 years and 75+ years) and TNM-stage (Sobin and Fleming, 1997). This provides insight on the role of breast cancer screening in trends of breast cancer among survivors.

## Results

Within 10 years of the first cancer diagnosis, 100 of 3368 (3%) and 182 of 5507 (3%) female cancer survivors diagnosed in the 1970s and in the 1990s, respectively, were subsequently diagnosed with breast cancer. Compared to patients diagnosed in the 1970s, cancer survivors diagnosed with a first cancer in the 1990s were 2.1 years older at first diagnosis and were more likely to have received systematic treatment (combined with either surgery or radiotherapy; Table 1). In both periods, digestive and urogenital cancers were the most common first non-breast cancer tumours.

Compared with the first period, a 30% increase in breast cancer incidence was observed in female cancer survivors (age-adjusted rate ratio (RR-adj): 1.30; 95% CI: 1.03–1.68; Table 2). Most marked increase in incidence of breast cancer was found among women previously diagnosed with non-breast cancer (RR-adj: 1.41; 95% CI: 1.04–2.75). There appeared to be a similar increase in breast cancer incidence across most treatment groups, albeit with wide confidence intervals. After adjusting for age, treatment and type of first cancer, the increase over time in breast cancer incidence among survivors became larger (RR-adj: 1.42; 95% CI: 1.11–1.83). This corresponded to a 201 excess cases per 100 000 female cancer survivors.

Table 3 shows that the increase over time in the incidence of breast cancer among female cancer survivors was similar for all age groups. The incidence rate tripled for stage II breast cancer (RR-adj: 3.10; 95% CI: 1.73–5.78) between the late 1970s and the early 1990s.

**Figure 1:**
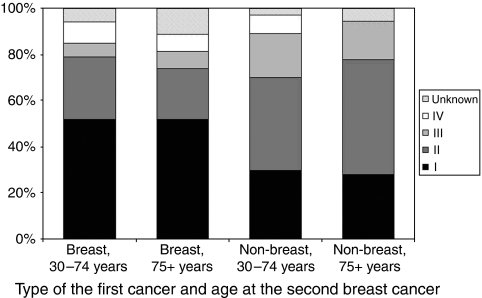
Stage distribution of breast cancer in female cancer survivors diagnosed with a first cancer in 1990–1994 according to type of first cancer and age at (second) breast cancer diagnosis.

Figure 1 illustrates the stage distribution of breast cancer for female survivors diagnosed with a first cancer in 1990–1994 categorised according to age at second breast cancer diagnosis and type of first cancer. A significant difference in stage distribution was found for the non-breast cancer survivors compared to those with a previous breast cancer. The proportion of second breast cancer stages II and III among non-breast cancer survivors was 62%, compared to only 32% among breast cancer survivors (*P* value=0.005).

## Discussion

In the general population, a 20% increase in the incidence of breast cancer has been reported over a 15-year period. (Louwman *et al*, 2008) In the same period, we observed an increase of 30% in the incidence of breast cancer among female cancer survivors. This increase was largest for non-breast cancer survivors and for second breast cancer stage II. The relatively large increase in breast cancer incidence among cancer survivors may be due to several factors: (1) The breast cancer early detection program (mass screening) that started in the early 1990s in the Netherlands; (2) application of more combined therapies with higher carcinogenic potential and (3) lifestyle, reproductive and hormonal factors, such as a longer interval between menarche and date of first birth, use of hormonal replacement therapy and alcohol use.

In the early 1990s, biennial mass screening for breast cancer was implemented for all women aged 50–69 years. In 1998, this program was expanded to include women up to the age of 75 (Verbeek and Broeders, 2003). Due to the intensified use of mammography for mass screening, the incidence of breast cancer increased by about 30% (Fracheboud *et al*, 2004) and may thus also be responsible for the increased incidence of breast cancer among cancer survivors. Consistently, we observed a significant increase in the proportion of female survivors diagnosed with early breast cancer (stages I and II being 54% in 1970s *vs* 76% in 1990s). On the other hand, we observed a similar increase in the incidence of breast cancer among survivors that do not fall in the screening age-target group (<50 and >74 years) to those targeted by screening (50–74 years). Furthermore, the increase of breast cancer incidence among female survivors is larger than that of the general population. This suggests that screening cannot fully account for this increase. Other breast cancer risk factors are likely to have contributed to this increase, including older age at first childbirth, fewer children, alcohol and other determinants of post-menopausal obesity (Althuis *et al*, 2005).

In the Netherlands, patients with breast cancer receive enhanced surveillance: annual mammography until the age of 60, followed by a biennial mammography up to the age of 74 (NABON Nationaal Borstkanker Overleg Nederland, 2005). This intense surveillance pattern is likely to explain the better stage distribution compared with the non-breast cancer survivors. In addition, it may also contribute to an increase in detection rates for slow-growing tumours that would have remained in the pre-clinical phase longer without screening mammography (Shen *et al*, 2005). Thus, breast cancer survivors are probably diagnosed more often and earlier with a low-grade breast cancer, which may require less aggressive treatment than other cancers (Moody-Ayers *et al*, 2000).

Changes in therapy for first primary cancer may have influenced the incidence of second breast cancer over time. Radiotherapy has been associated with an approximately 40% higher risk of developing subsequent breast cancer compared to systemic hormonal or cytotoxic treatment (Soerjomataram *et al*, 2005; Schaapveld *et al*, 2008). In addition, cancer patients who receive a high radiation dose on the chest as part of treatment have higher breast cancer risk. For example, young women with Hodgkin lymphoma may have up to 29% cumulated risk of breast cancer 30 years after treatment. (Travis *et al*, 2005) On the other hand, radiotherapy treatment techniques and protection of the normal tissue have improved in the last decades. In our study, we observed a similar increase in the incidence of breast cancer over time for those who were irradiated and those who only underwent surgery. The increasing incidence of breast cancer among those who were irradiated could therefore not be attributed to radiotherapy. However, because we followed patients for only 10 years, we may have missed late adverse effects of radiation (Travis, 2006). Although the use of systemic cancer treatment has doubled during the last 20 years, we found that the increase in the incidence of breast cancer among those who received systemic treatment was similar to surgically treated patients, whereas a decrease in incidence was expected. Studies have shown that cancer patients who have hormonal therapy (eg tamoxifen) or chemotherapy have a reduced risk of breast cancer (van Leeuwen *et al*, 2003; Early Breast Cancer Trialists’ Collaborative_Group, 2005). A protective effect of tamoxifen on second breast cancer is most evident in the post-treatment period (Powles *et al*, 2007). Thus, it is possible that changes in rates over time may differ for different categories of follow-up, for example, decreasing trend of breast cancer in survivors who received tamoxifen, which is only observable 8 years after treatment. Unfortunately, we did not have sufficient power to examine trends across different follow-up periods.

In view our results the following groups may need a more intensive follow-up for breast cancer. Firstly, non-breast cancer survivors. These patients had a larger proportion of stage II breast cancer than patients who had breast cancer as a first primary cancer. Among non-breast cancer patients diagnosed in 1990–1994 and followed up for 10 years, 29% of second breast cancers was stage 1, 44% stage 2, 18% stage III and 5% stage IV. For breast cancer patients, the corresponding percentages were 52% for stage I, 26% for stage II, 6% for stage III and 9% for stage IV (*P* value of *χ*^2^: 0.005). Thus, although the absolute risks (2% during 10-year follow-up) remain small, female survivors of non-breast cancer would probably benefit from a more intensive follow-up than just the mass-screening program, such as an additional biennial clinical breast examination. (Rijnsburger *et al*, 2004) A second group is female survivors older than 75 years. (Boer *et al*, 1995) Survivors of this age group exhibited the largest increase in breast cancer incidence over time. Furthermore, compared to the general population, they had a threefold higher risk of breast cancer than the general population. (Comprehensive_Cancer_Centre, 2007) Given a worse stage distribution especially among those diagnosed with a non-breast cancer, it seems logical to extend the screening program to include older female survivors. However, mortality due to other causes is high (Hooning *et al*, 2006) and the existence of comorbidities would probably limit treatment choices. (Louwman *et al*, 2005) Furthermore, early detection increases overdiagnosis and may lead to overtreatment. In older women, this issue is greater due to the longer sojourn time of breast cancer, that is longer time in which a cancer is still asymptomatic, but already detectable by a screening test. (Fracheboud *et al*, 2006) Thus, a detailed cost-effectivity study, preferably adjusting for Quality of Life, is warranted. A last group is cancer survivors younger than 50 years. Although the incidence of breast cancer has not increased much over time, incidence was highest for survivors aged 30–49 years. This group may merit the same screening regimen as women with a genetic predisposition towards breast cancer, that is with an MRI.

It should be warranted that besides the apparent beneficial effect that is detecting cancer at an early stage, screening has also harmful effects. Therefore, a decision to screen should always weigh the benefit, harm and cost. (Fracheboud *et al*, 2006) The main unfavourable effect is overdiagnosis, that is diagnosis of cancers that would not have been detected if there had not been screening. This leads to a loss of quality of life, because the person has to live with a cancer diagnosis and unnecessary treatment. Furthermore, early detection using mammography may increase the lifetime exposure to radiation and hence the breast cancer risk. However, simulation studies have shown that screening prevented more deaths from breast cancer than it induced. (Beemsterboer *et al*, 1998).

In summary, we found a significant increase in the incidence of breast cancer among female cancer survivors, especially for non-breast cancer survivors. The increase in second breast cancers was most striking for stage II cancers. Our findings suggest that there is ample room for improvement in follow-up strategies to detect breast cancer at an early stage in this group.

## Figures and Tables

**Table 1:** Characteristics of female cancer survivors diagnosed in 1975–1979 and in 1990–1994 with a 10-year follow-up

Period of first primary cancer diagnosis	1975–1979		1990–1994	
Number of cancer survivors	3368		5507	
Women-years of follow-up	19 287		26 913	
Mean age at first cancer (years)	60.5		62.7	
Mean follow-up time (years)	5.7		4.9	
				
	***N***	**%**	***N***	**%**
*Age at diagnosis of first cancer*				
30–49 years	791	23	1200	22
50–74 years	2070	61	3086	56
75+ years	507	15	1221	22
				
*Treatment of first cancer*				
Surgery	1417	42	2248	41
Radiotherapy±surgery	1245	37	1450	26
Systemic therapy±surgery	322	10	767	14
Radiotherapy+systemic therapy±surgery	220	6	760	14
No therapy	164	5	282	5
				
*Type of first cancer*				
Breast cancer	1436	43	2199	40
Non-breast cancer^a^	1932	57	3308	60

*N* indicates number of cases and % indicates column's percentage.

a First non-breast cancer in 1970s consisted of 3% respiratory cancers, 40% digestive cancers, 34% urogenital cancers (of which 24% (155 cases) were ovarian cancer cases), 9% haematopoietic cancers (Hodgkin lymphoma 19 cases) and 15% other cancers, and in 1990s 7% respiratory cancers, 33% digestive cancers, 30% urogenital cancers (of which 24% (238 cases) were ovarian cancer cases), 10% haematopoietic cancers (Hodgkin lymphoma 17 cases) and 19.3% other cancers.

**Table 2:** Number (*N*) and age-standardised incidence rates of breast cancer per 100 000 women-years for female cancer survivors^a^

	**1970s**		**1990s**			
**Period of first primary cancer diagnosis**	***N***	**Incidence (100 000)**	***N***	**Incidence (100 000)**	**Rate ratio^b^**	**(95% CI)**
*Type of first primary cancer* c						
Breast cancer	77	839	127	999	1.19	0.91–1.60
Non-breast cancer	23	228	55	387	1.41	1.04–2.75
						
*Treatment of first primary cancer* c						
Surgery	39	451	74	634	1.41	0.95–2.08
Radiotherapy±surgery	48	625	64	851	1.36	0.94–1.98
Systemic therapy±surgery	5	371	15	520	1.40	0.48–3.75
Radiotherapy+systemic therapy±surgery	7	702	25	613	0.87	0.39–2.10
No therapy	1	157	4	531	3.38	0.40–31.69
Incidence rates (only age adjusted)^c^	100	519	182	676	1.30	1.03–1.68
Incidence rates (age, type of first cancer and treatment of first cancer adjusted)	100	475	182	676	1.42	1.11–1.83

*N* indicates number of cases.

a Female cancer survivors are both breast cancer and non-breast cancer survivors.

b Rate ratio compares incidence rate in 1990s with incidence rate in 1970s.

c Age adjusted.

**Table 3:** Incidence rates of breast cancer per 100 000 women-years for female cancer survivors^a^ according to age and stage of breast cancer (second primary) in 1970s and 1990s

	**1970s**		**1990s**			
**Period of first primary cancer diagnosis**	**60.8**		**63.2**			
**Mean age at breast cancer (years)**	***N***	**Incidence (100 000)**	***N***	**Incidence (100 000)**	**Rate ratio^b^**	**(95% CI)**
*Age at breast cancer (second primary – age-specific rates)*						
30–49 years	23	678	43	887	1.3	0.8–2.2
50–74 years	61	509	94	615	1.2	0.9–1.7
75+	16	409	45	664	1.6	0.9–2.9
						
*Stage of breast cancer (second primary)* c						
I	41	209	82	305	1.46	0.99–2.11
II	13	68	57	212	3.10	1.73–5.78
III	11	58	18	67	1.16	0.56–2.50
IV	9	46	14	52	1.14	0.50–2.65
Unknown	26	140	11	41	0.29	0.14–0.59

*N* indicates number of cases.

a Female cancer survivors are both breast cancer and non-breast cancer survivors.

b Rate ratio compares incidence rate in 1990s with incidence rate in 1970s, if adjusted rates are presented than rate ratio is based on the standardised rates.

c Age adjusted.
